# Cytogenetic data on *Ancistrus* sp. (Siluriformes, Loricariidae) of the Paraguay River basin (MS) sheds light on intrageneric karyotype diversification

**DOI:** 10.3897/CompCytogen.v10i4.8532

**Published:** 2016-11-22

**Authors:** Ana Camila Prizon, Luciana Andreia Borin-Carvalho, Daniel Pacheco Bruschi, Marcos Otávio Ribeiro, Ligia Magrinelli Barbosa, Greicy Ellen de Brito Ferreira, Andréa Cius, Claudio Henrique Zawadzki, Ana Luiza de Brito Portela-Castro

**Affiliations:** 1 Departamento de Biotecnologia, Genética e Biologia Celular, Universidade Estadual de Maringá, UEM, Avenida Colombo, 5790, 87020-900, Maringá, Paraná, Brazil; 2 Departamento de Biologia/ Nupélia, Universidade Estadual de Maringá, UEM, Avenida Colombo, 5790, 87020-900, Maringá, Paraná, Brazil; 3 Departamento de Genética, Universidade Federal do Paraná, UFPR, Centro Politécnico, Jardim Botânico, 81531-980, Curitiba, Paraná, Brazil

**Keywords:** Ancistrini, cytotaxonomy, CMA_3_/DAPI, heterochromatin, rDNA

## Abstract

*Ancistrus* Kner, 1854 is a diverse catfish genus, currently comprising 66 valid species, but karyotype data were recorded for 33 species, although only ten have their taxonomic status defined. Considerable karyotype diversity has been found within this genus, with 2n varying from 34 to 54 and structural variability including heteromorphic sex chromosomes. In many cases, uncertainty on the taxonomic status of the study populations hampers reliable interpretation of the complex chromosomal evolutionary history of the group. This study aims to present the first karyotype data for a population of the *Ancistrus* sp. collected in Criminoso stream (tributary of the Paraguay River Basin, Mato Grosso do Sul, Brazil) in which a combination of different chromosomal markers was used and results integrated in broad discussion on karyotype evolution in the genus. The specimens presented 2n=42 with 18m+16sm+8st and a single NOR revealed by silver nitrate and fluorescence *in situ* hybridization (FISH) with 18S rDNA probe, located in pair No. 10. Clusters of 5S rDNA were located in the pericentromeric region of three chromosomes: pair No. 1 (metacentric) and one of the homologues of the nucleolar pair No. 10. Heterogeneity in the molecular composition of the heterochromatin was confirmed by the association of C-banding and fluorochrome CMA_3_/DAPI-staining. Exploring the differential composition of constitutive heterochromatin in *Ancistrus* may provide an important perspective to understand genome organization and evolution within this group. Our data reinforce the chromosomal diversity present in *Ancistrus* genus and we discuss the potential sources these variation. The karyotype structure of *Ancistrus* sp. “Criminoso stream” appears to be consistent with the existence of a new candidate species.

## Introduction

*Ancistrus* Kner, 1854 is the most species-rich genus of the tribe Ancistrini (Hypostominae), currently consisting of 66 valid species (Bifi et al. 2009, Froese and Pauly 2016). This genus is widespread in the Neotropical region, from Panama to Rio de La Plata in Argentina, although the greatest diversity of *Ancistrus* species is found in the basin of the Amazon River (Fisch-Muller 2003).

Up until now, the karyotypes of 33 *Ancistrus* species have been described, even though most of these species have yet to be formally identified (e.g. de Oliveira et al. 2009, Mariotto et al. 2011, 2013, Favarato et al. 2016). The karyotype data available for this genus indicate considerable chromosomal diversity, with diploid numbers ranging from 2n=34 chromosomes in *Ancistrus cuiabae* (Mariotto et al. 2009) to 2n=54 in *Ancistrus claro* (Mariotto et al. 2011). In addition to this numerical diversity, there is considerable variation in chromosome structure, including differences among populations that suggest the existence of species complexes, as observed in Ancistrus prope dubius (Mariotto and Miyazawa 2006). Another remarkable feature of *Ancistrus* is the occurrence of heteromorphic sex chromosomes in some species, including simple (Mariotto et al. 2004, Alves et al. 2006, Mariotto and Miyazawa 2006) and multiple systems (de Oliveira et al. 2007, 2008) which has also contributed to karyotype diversification within the genus.

The chromosomal mapping of the two classes of rDNA (45S and 5S genes) has contributed to the understanding of the organization and evolutionary dynamics of these genes in fish genomes. The 5S rDNA sites are commonly located in interstitial or proximal positions and separated from the 45S rDNA genes, as observed in many groups, but in Loricariidae little is known about the distribution and number of 5S ribosomal genes, being more studied for some species of *Hypostomus* Lacépède, 1803 (Kavalco et al. 2004, 2005, Mendes-Neto et al. 2011, Traldi et al. 2012, Bueno et al. 2014) and some *Ancistrus* species (Mariotto et al. 2011, Konerat et al. 2015, Favarato et al. 2016). These studies indicate that the distribution of 5S rDNA genes varies considerably and may occur on one or more chromosome pairs, with or without synteny among the 18S rDNA sites.

The complex taxonomic scenario that has been noted in the *Ancistrus* genus also contributes to the difficulty in understanding the karyotype evolution of this group. In the present study, we provide a detailed description of the karyotype of *Ancistrus* sp. “Criminoso stream” based on specimens collected in the basin of the Paraguay River (Brazil), using classical and molecular cytogenetic techniques. We provide physical chromosome maps of the 18S and 5S rDNA clusters, and of the heterochromatin, highlighting the GC- and AT-rich composition using base-specific fluorochromes. We also compiled the cytogenetic data available for the genus *Ancistrus* to provide a more systematic overview of the karyotypic variation in this group.

## Material and methods

Authorization for the collection of specimens was granted by the Brazilian Environment Ministry through its Biodiversity Information and Authorization System (SISBIO), under the license number 36575-1. The protocols used in this study were submitted and reviewed by the Ethics Committee on the use of animals (CEUA) of Universidade Estadual de Maringá under the case number 013/2009.

Cytogenetic analyses were conducted on 13 specimens (5 females, 6 males and 2 of undetermined sex) of *Ancistrus* sp. collected from the Criminoso stream (18°29.333’S, 54°45.233’W), a small tributary of the Taquari River, near the town of Coxim in the basin of the upper Paraguay River, in Mato Grosso do Sul state, Brazil. The specimens were identified as *Ancistrus* sp. “Criminoso stream” (NUP 12018) and deposited in the ichthyological collection of the Limnology, Ichthyology and Aquaculture Research Center (Nupélia) at Maringá State University, Paraná, Brazil.

Chromosome preparations were obtained from kidney cells following the technique described by Bertollo et al. (1978). The nucleolus organizer regions (NOR) were detected by impregnation with silver nitrate (Ag-NO_3_), as described by Howell and Black (1980). Double staining was carried out with chromomycin A_3_ (CMA_3_) and DAPI, according to Schweizer (1976). The constitutive heterochromatin was identified by the C-banding technique as described in Sumner (1972) and stained with propidium iodide according to the method of Lui et al. (2012). The physical mapping of the 18S and 5S rDNA sequences was carried out by Fluorescence *in situ* Hybridization (FISH) according to Pinkel et al. (1986). The 18S probe was obtained from *Prochilodus argenteus* Spix et Agassiz, 1829 (Hatanaka and Galetti Jr. 2004), and labelled with the Nick Translation Biotin kit and 5S rDNA probes from *Leporinus elongatus* Valenciennes, 1850 (Martins and Galetti Jr. 1999) labelled with the Nick Translation Digoxigenin kit. The hybridization signals were detected using avidin-FITC (fluorescein isothiocyanate) for the 18S rDNA probe and anti-digoxigenin-rhodamine for the 5S rDNA probe. The chromosomes were counterstained with DAPI. The metaphases were photographed using an epifluorescence microscope and optimized for best contrast and brightness with Adobe Photoshop CS6 software.

The chromosomes were identified based on the modified arm ratio (AR) criteria of Levan et al. (1964), and classified as metacentric (m), submetacentric (sm), subtelocentric (st) and acrocentric (a). The fundamental number (FN) was established considering the meta-submetacentric and subtelocentric chromosomes to have two arms and the acrocentric chromosomes, only one.

## Results

**Figure 1.**
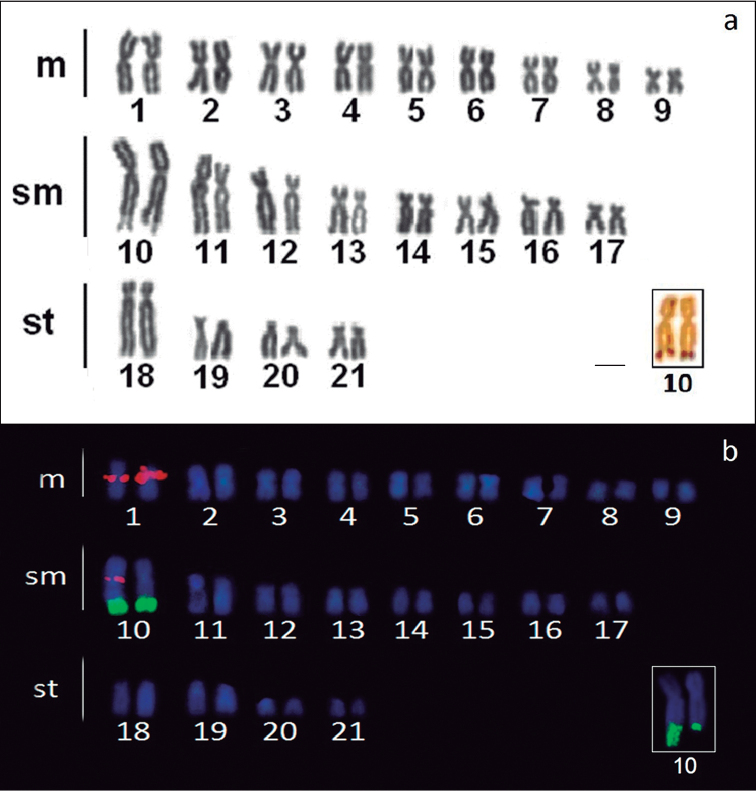
Karyotype of *Ancistrus* sp. “Criminoso stream” after: **a** Giemsa-staining and the NOR-bearing chromosome pair No. 10 (in box) **b** double-FISH using 18S rDNA (green) and 5S rDNA (red) probes. Note the size heteromorphism in the NOR-bearing chromosomes from a different metaphase (in box). Bar = 10 µm.

The specimens of *Ancistrus* sp. “Criminoso stream” had a diploid number of 2n=42 with 18m+16sm+8st and a FN of 84, in both sexes (Figure 1a). The Ag-NOR sites were found in the terminal position of the long arm of a submetacentric pair (No. 10), which presented a clear size heteromorphism (Figure 1a, in box), also confirmed by 18S rDNA-FISH (Figure 1b, in box). The 5S rDNA sites were detected in the pericentromeric regions of the first pair of metacentric chromosomes and in one of the homologues of the nucleolar pair (Figure 1b).

**Figure 2.**
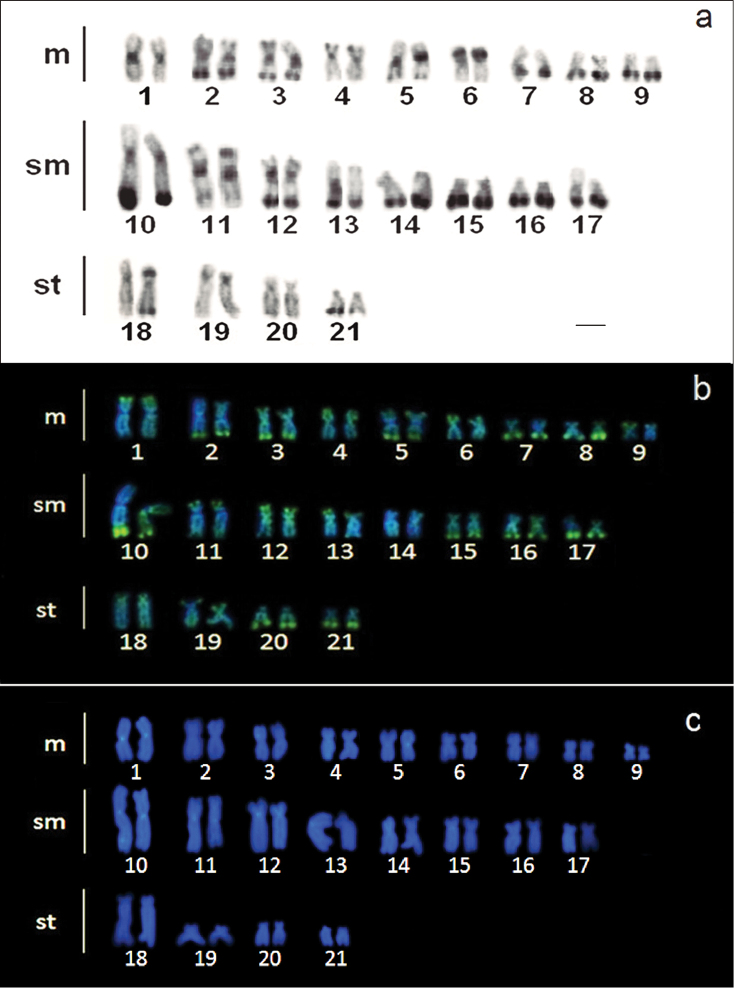
Karyotype of *Ancistrus* sp. “Criminoso stream” showing: **a** the heterochromatin distribution pattern after C-banding **b** CMA_3_/DAPI base-specific profile and **c** DAPI staining. Bar = 10 µm.

**Figure 3.**
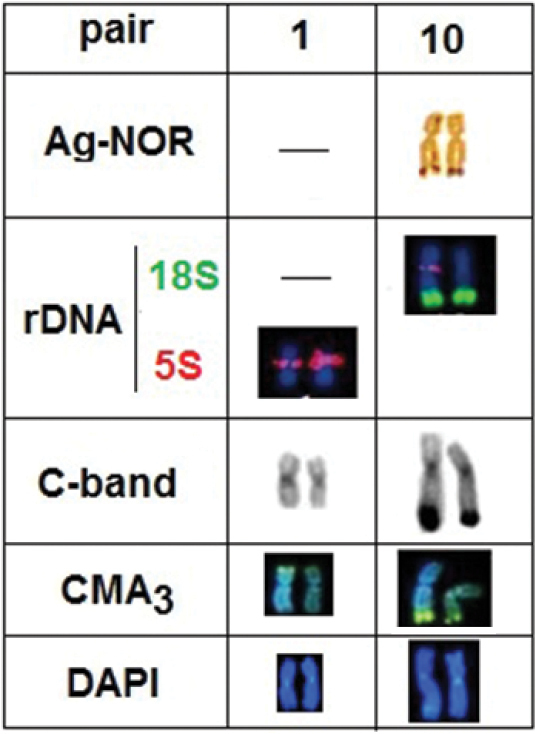
Chromosome pairs, 1 and 10 of *Ancistrus* sp “Criminoso steam” after different banding: pair No 1 showing the localization of the pericentromeric 5S rDNA sites (red), positive for C-band and DAPI; pair No 10 showing Ag-NOR sites coincident with 18S rDNA signals (green), positive for C-band and CMA_3_; pericentromeric 5S rDNA sites (red) were shown in only one of the homologues of the pair 10, whose sequence was coincident with heterochromatic blocks (C-band) and DAPI in this pair.

C-banding detected telomeric and pericentromeric heterochromatic blocks in almost all chromosomes pairs of *Ancistrus* sp. “Criminoso stream” (Figure 2a). The chromosome locations corresponding to 5S (pair No. 1 and one homologue of pair No. 10) and 18S rDNA (pair No. 10) were also positive for C-bands. Some of the chromosomes also presented interstitial heterochromatic blocks (pairs Nos. 2, 3, 6, 9 and 11). Chromomycin A_3_ staining revealed several GC-rich regions besides the Ag-NORs sites (Figure 2b), which coincided with most of the heterochromatic blocks in the telomeric regions (Figure 2a). The DAPI fluorochrome produced a brighter signal in the centromeric regions of chromosome pairs Nos. 1, 5, 10, 11, 12 and 13 (Figure 2c). It should be noted that these DAPI^+^ blocks in pairs Nos. 1 and 10 are interspersed with the 5S rDNA sites (Figures 1b, 2c). These heterochromatin blocks were observed in all specimens analyzed, irrespective of the sex. Figure 3 highlights the pairs 1 and 10 with all banding.

## Discussion

The karyotypes described for the genus *Ancistrus* have been obtained from species found in the basins of the Paraguay and Amazon Rivers, with extensive variability observed in the genus, whose diploid numbers vary of 34, 38, 40, 42, 44, 46, 48, 50, 52 and 54 chromosomes (de Oliveira et al. 2009, Mariotto et al. 2011, 2013, Favarato et al. 2016). The available descriptions of karyotypes for unidentified *Ancistrus* species emphasize the potential diversity of the genus in these basins. The 2n=42 karyotype as observed in *Ancistrus* sp. “Criminoso stream” has also been recorded in other *Ancistrus* species albeit with distinct karyotype formulae (Table 1), indicates the presence of another species, yet to be identified, in the Pantanal region. Additional taxonomic studies are clearly needed to determine the full diversity of the genus in the region.

**Table 1.** Cytogenetic data available for *Ancistrus* with 2n=42.

Species	River/Basin/ State	2n	NF	Karyotype formulae/Sex chromosome	NOR		rDNA 5S	Ref
					Ag-NOR	rDNA18S		
Ancistrus cf. dubius	Pantanal/Paraguay/ MT	44	80	18m+10sm+8st+8a / ZZ/ZW	it	sm, it (16)*	sm, it (16)*; m, it (4); sm, pc (14)	Mariotto et al. 2004, 2006, 2011
		42	84	24m+10sm+8st / XX/XY	it			
		42	84	24m+10sm+8st	it			
*Ancistrus* sp. 12	Santa Cruz/ Paraguay/MT	42	84	28m+10sm+4st	it	–	–	Mariotto 2008
*Ancistrus* sp. 10	Vermelho/Paraguay/MT	42	82	22m+14sm+4st+2a / ZZ/ZW	it	–	–	Mariotto 2008
*Ancistrus* sp. 11	Araputanga/ Paraguay/MT	42	84	24m+12sm+6st / XX/XY	it	–	–	Mariotto 2008
*Ancistrus* sp. Vermelho	Demini/Amazon/AM	42	78	26m+6sm+4st+6a	te	a, (20)	–	de Oiveira et al. 2009
*Ancistrus* sp. “Criminoso stream”	Criminoso stream/Paraguay/MS	42	84	18m+16sm+8st	te	sm, te (10)*	sm, it (one chromosome, 10)*; m, it (1)	Present study

Subtitles: 2n : diploid number ; NF : fundamental number ; m : metacentric ; sm : submetacentric ; st : subtelocentric ; a : acrocentric ; it : interstitial ; te : terminal ; pc : pericentromeric ; – : not registered ; * : synteny between 18S and 5S rDNA sites ; numbers in parentheses refer to the chromosome pairs; MT : Mato Grosso ; AM : Amazonas ; MS : Mato Grosso do Sul ; Ref : references .

The karyotype data available for *Ancistrus* indicate a relation between diploid number and chromosome types; species with a 2n=44 or more have a larger number of acrocentric chromosomes, while species with smaller diploid numbers (2n=34 to 42) have few or no acrocentric chromosomes (de Oliveira et al. 2009, Medeiros et al. 2016). Artoni and Bertollo (2001) proposed that a karyotype of 2n=54 with a predominance of meta-submetacentric pairs was the primitive condition for the family Loricariidae, which was thus far been found in *Ancistrus* sp. 01, *Ancistrus* sp. 03 (Mariotto et al. 2013) and *Ancistrus claro* (Mariotto et al. 2011) species, with karyotype formula of 14m+8sm+8st+24a. These species have a proportion karyotype of 22 meta-submetacentric (40.7%) and 32 st-acrocentric chromosomes (59.3%). From this starting point, there has been an extensive reduction in the diploid number in the genus *Ancistrus* (54 to 34) due to possible chromosomal fusions, which can be deduced from the inverse relationship between the number of acrocentric and meta-submetacentric chromosomes. This can be seen in the species with reduced diploid numbers (40, 38, 34), which show little or no acrocentric chromosomes (Mariotto et al. 2004, 2006, 2009, 2011, de Oliveira et al. 2009, Favarato et al. 2016, Medeiros et al. 2016). However, other rearrangements such as inversions and/or translocations also occurred during the evolution of the karyotype, resulting in the considerable diversity of the fundamental number observed in this genus

Chromosome banding in *Ancistrus* sp. “Criminoso stream” revealed a single nucleolar pair (Ag-NOR), a character shared with all other species of the genus analyzed to date, except *Ancistrus* sp. (Reis et al. 2012). This arrangement has been confirmed by FISH of the 18S rDNA in some species. A single NOR in an interstitial location has been considered the plesiomorphic condition in Loricariidae (Artoni and Bertollo 2001, Alves et al. 2003, de Oliveira et al. 2009, Mariotto et al. 2009, 2011, Mendes-Neto et al. 2011). However, in *Ancistrus*, the NOR has been observed in a terminal position for most species but, there is also a significant number of species with interstitial NORs (Medeiros et al. 2016). The structural diversity of NORs in *Ancistrus* related to the different types and sizes of chromosomes may be associated with extensive structural rearrangements (fusions, translocations and/or inversions) that occurred during the karyotype diversification of the genus (de Oliveira et al. 2009, Mariotto et al. 2011). A clear example of these rearrangements was provided by Mariotto et al. (2009) in *Ancistrus cuiabae*, which has a pericentric inversion in the nucleolar pair (Ag-NOR) supported by C-banding and 18S rDNA-FISH.

Assuming that the primitive Loricariidae karyotype was composed of 2n=54 chromosomes, with synteny between the 18S and 5S rDNA sites as in *Ancistrus claro*, Mariotto et al. (2011) suggested that this layout may represent the basal condition for the genus. This proposal was supported for the species *Ancistrus dolichopterus*, *Ancistrus* prope *dolichopterus*, *Ancistrus ranunculus* and *Ancistrus maximus* analyzed by Favarato et al. (2016). Thus, *Ancistrus* sp. “Criminoso stream” presented a derived condition related to synteny break between the rDNA 18S and 5S sites (pairs 10 and 1, respectively), but the presence of an interstitial 5S rDNA site in one homologues of nucleolar (pair No. 10), may be a remnant of the primitive condition in this genus.

The heterogeneity in the molecular composition of the heterochromatin of *Ancistrus* sp. “Criminoso stream” was demonstrated by the combination of C-banding, CMA_3_ and DAPI-staining, representing a valuable approach in comparative cytogenetics. DAPI bands were clearly related with the pericentromeric heterochromatin observed by C-banding in most chromosomes and coincided with the 5S rDNA sites (pair No. 1 and one homologue of pair No. 10). The GC-rich heterochromatin associated with (or interspersed between) ribosomal genes, as observed, is common in most fish chromosomes; however, the presence of terminal CMA_3_^+^/C-band^+^ blocks in most chromosomes is not a feature frequent in fishes karyotype, included *Ancistrus* species. In addition, to date there is no karyotypic study in *Ancistrus* demonstrating coincidence of heterochromatin blocks in several chromosomes with GC/AT rich content, as obtained in the species under study. So, it could be a good chromosomal mark to characterize the karyotype of this putative new species. All *Ancistrus* species cytogenetically described to date exhibit profiles of low constitutive heterochromatin content, with varied distribution form being found in interstitial and pericentromeric regions to occupying large portions of the long or short chromosome arms (e.g. de Oliveira et al. 2009). In some cases, the accumulation of heterochromatin could be related to the origin of heteromorphic sex chromosomes, as found in *Ancistrus* prope *dubius* (Mariotto et al. 2004, Mariotto and Miyazawa 2006) and *Ancistrus taunayi* (Konerat et al. 2015), being also associated with paracentric inversions in the latter. Particularly in *Ancistrus* sp. “Criminoso stream” we hypothesized that unique constitutive heterochromatin pattern observed in their karyotype sheds light on intrageneric karyotype diversification, however such studies are needed in other species of the genus.

## Conclusion

Our results further reinforce the considerable variation in karyotype macrostructure within the genus through the description of a new karyotype formula and unique constitutive heterochromatin pattern observed in the population of the *Ancistrus* sp. “Criminoso stream”, contributing with information about the complex chromosomal evolution history of the catfish genus.

This result also appears to be consistent with the existence of a new candidate species. Notwithstanding, our results also emphasize the need for an integrated approach to the understanding of the taxonomic status of this population, based on morphological, ecological, and molecular data. Ultimately, ecological and behavioral traits other than reproductive strategies may be contributing to the mechanisms of isolation that underpin the chromosomal diversification in *Ancistrus*.

## References

[B1] Alves AL Oliveira C Foresti F (2003) Karyotype variability in eight species of the subfamilies Loricariinae and Ancistrinae (Teleostei, Siluriformes, Loricariidae). Caryologia 1: 57–63. doi: 10.1080/00087114.2003.10589308

[B2] Alves AL Oliveira C Nirchio M Granado A Foresti F (2006) Karyotypic relationships among the tribes of Hypostominae (Siluriformes: Loricariidae) with description of XO sex chromosome system in a Neotropical fish species. Genetica 128: 1–9. doi: 10.1007/s10709-005-0715-1 17028935 10.1007/s10709-005-0715-1

[B3] Artoni RF Bertollo LAC (2001) Trends in the karyotype evolution of Loricariidae fish (Siluriformes). Hereditas 134: 201–210. doi: 10.1111/j.1601-5223.2001.00201.x 11833282 10.1111/j.1601-5223.2001.00201.x

[B4] Bertollo LAC Takahashi CS Moreira Filho O (1978) Cytotaxonomic considerations on *Hoplias lacerda* (Pisces, Erythrinidae). Brazilian Journal of Genetics 1: 103–120.

[B5] Bifi A Pavanelli GCS Zawadzki CH (2009) Three new species of *Ancistrus* Kner, 1854 (Siluriformes: Loricariidae) from the Rio Iguaçu basin, Paraná State, Brazil. Zootaxa 2275: 41–59.

[B6] Bueno V Venere PC Konerat JT Zawadzki CH Vicari MR Margarido VP (2014) Physical mapping of the 5S and 18S rDNA in ten species of *Hypostomus* Lacépède (Siluriformes: Loricariidae): evolutionary tendencies in the genus. The Scientific World Journal 2014: 1–8. doi: 10.1155/2014/943825 10.1155/2014/943825 PMC4227443 25405240

[B7] Favarato RM Silva M de Oliveira RR Artoni RF Feldberg E Matoso DA (2016) Cytogenetic Diversity and the Evolutionary Dynamics of rDNA Genes and Telomeric Sequences in the *Ancistrus* Genus (Loricariidae: Ancistrini). Zebrafish 13: 103–111. doi: 10.1089/zeb.2015.1140 26829587 10.1089/zeb.2015.1140

[B8] Froese R Pauly D (2016) FishBase: World Wide Web electronic publication. http://www.fishbase.org [accessed 08. August 2016]

[B9] Fisch-Muller S (2003) Subfamily Ancistrinae. (armored catfishes). In: Reis RE Kullander SO Ferraris CJ Jr (Eds) Check list of the freshwater fishes of South and Central America. Porto Alegre, 373–400.

[B10] Hatanaka T Galetti PM Jr (2004) Mapping 18S and 5S ribosomal RNA genes in the fish *Prochilodus argenteus* Agassiz, 1929 (Characiformes, Prochilodontidae). Genetica 122: 239–244. doi: 10.1007/s10709-004-2039-y 15609546 10.1007/s10709-004-2039-y

[B11] Howell WM Black DA (1980) Controlled silver-staining of nucleolus organizer regions with a protective colloidal developer: a 1-step method. Experientia 36: 1014–1015. doi: 10.1007/BF01953855 6160049 10.1007/BF01953855

[B12] Kavalco KF Pazza R Bertollo LAC Moreira-Filho O (2004) Heterochromatin characterization of four fish species of the family Loricariidae (Siluriformes). Hereditas 141: 237–242. doi: 10.1111/j.1601-5223.2004.01850.x 15703039 10.1111/j.1601-5223.2004.01850.x

[B13] Kavalco KF Pazza R Bertollo LAC Moreira-Filho O (2005) Karyotypic diversity and evolution of Loricariidae (Pisces, Siluriformes). Heredity 94: 180–186. doi: 10.1038/sj.hdy.6800595 15562288 10.1038/sj.hdy.6800595

[B14] Konerat JT Bueno V Margarido VP Portela-Castro ALB Martins-Santos IC (2015) Diversity of Sex Chromosome Systems in Ancistrini (Loricariidae, Hypostominae): ZZ/ZW in *Ancistrus taunayi* Miranda Ribeiro, 1918. Cytogenetic and Genome Research 146: 306–310. doi: 10.1159/000441431 26523437 10.1159/000441431

[B15] Levan A Fredga K Sandberg AA (1964) Nomenclature for centromeric position on chromosomes. Hereditas 52: 201–220. doi: 10.1111/j.1601-5223.1964.tb01953.x

[B16] Lui RL Blanco DR Moreira-Filho O Margarido VP (2012) Propidium iodide for making heterochromatin more evident in the C-banding technique. Biotechnic & Histochemistry 87: 433–438. doi: 10.3109/10520295.2012.696700 22747174 10.3109/10520295.2012.696700

[B17] Martins C Galetti-Jr PM (1999) Chromosomal localization of 5S rDNA genes in *Leporinus* fish (Anostomidae, Characiformes). Chromosome Research 7: 363–367. doi: 10.1023/A:1009216030316 10515211 10.1023/a:1009216030316

[B18] Mariotto S Artoni RF Miyazawa CS (2004) Occurence of sexual chromosome, of the type ZZ/ZW, in Ancistrus cf. dubius (Loricariidae, Ancistrinae) of the Paraguay River Basin, Mato Grosso, Brazil. Caryologia 57: 327–331. doi: 10.1080/00087114.2004.10589413

[B19] Mariotto S Miyazawa CS (2006) Ancistrus cf. dubius (Siluriformes, Ancistrinae), a complex of species. 1. Chromosomal characterization of four populations and occurrence of sex chromosomes of the type XX/XY, in the Pantanal Basin of Mato Grosso, Brazil. Caryologia 59: 299–304. doi: 10.1080/00087114.2006.10797929

[B20] Mariotto S (2009) Estudo citogenético clássico e molecular em quinze espécies da tribo Ancistrini (Siluriformes, Loricariidae) de três bacias hidrográficas brasileiras. PhD Thesis, Universidade Federal de São Carlos, São Carlos, 101 pp. doi: 10.1590/S1679-62252009000400006 [In Portuguese]

[B21] Mariotto S Centofante L Miyazawa CS Bertollo LAC Moreira-Filho O (2009) Chromosome polymorphism in *Ancistrus cuiabae* Knaack, 1999 (Siluriformes: Loricariidae: Ancistrini). Neotropical Ichthyology 7: 595–600. doi: 10.1590/S1679-62252009000400006

[B22] Mariotto S Centofante L Vicari MR Artoni RF Moreira-Filho O (2011) Chromosomal diversification in ribosomal DNA sites in *Ancistrus* Kner, 1854 (Loricariidae, Ancistrini) from three hydrographic basins of Mato Grosso, Brazil. Comparative Cytogenetics 5: 289–300. doi: 10.3897/compcytogen.v5i4.1757 24260636 10.3897/CompCytogen.v5i4.1757 PMC3833787

[B23] Mariotto S Centofante L Moreira-Filho O (2013) Diversity and chromosomal evolution in the genus *Ancistrus* Kner, 1854 (Loricariidae: Ancistrini) from three hydrographic basins of Mato Grosso State, Brazil. Neotropical Ichthyology 11: 125–131. doi: 10.1590/S1679-62252013000100015

[B24] Medeiros LA Ginani EG Sousa LM Py-Daniel LHR Feldberg E (2016) Cytogenetic analysis of *Baryancistrus xanthellus* (Siluriformes:Loricariidae: Ancistrini), an ornamental fish endemic to the Xingu River, Brazil. Neotropical Ichthyology 14(2): e150108. doi: 10.1590/1982-0224-20150108

[B25] Mendes-Neto EO Vicari MR Artoni RF Moreira-Filho O (2011) Description of karyotype in *Hypostomus regain* (Ihering, 1905) (Teleostei, Loricariidae) from the Piumhi river in Brazil with comments on karyotype variation found in *Hypostomus*. Comparative Cytogenetics 5: 133–142. doi: 10.3897/compcytogen.v5i2.964 24260625 10.3897/compcytogen.v5i2.964 PMC3833738

[B26] de Oliveira RR Feldberg E Anjos MB Zuanon J (2007) Karyotype characterization and ZZ/ZW sex chromosome heteromorphism in two species of the catfish genus *Ancistrus* Kner, 1854 (Siluriformes: Loricariidae) from the Amazon basin. Neotropical Ichthyology 5: 301–306. doi: 10.1590/S1679-62252007000300010

[B27] de Oliveira RR Feldberg E Anjos MB Zuanon J (2008) Ocurrence of multiple sexual chromosomes (XX/XY_1_Y_2_ and Z_1_Z_1_Z_2_Z_2_/Z_1_Z_2_W_1_W_2_) in catfishes of the genus *Ancistrus* (Siluriformes, Loricariidae) from the Amazon Basin. Genetica 134: 243–249. doi: 10.1007/s10709-007-9231-9 18038220 10.1007/s10709-007-9231-9

[B28] de Oliveira RR Feldberg E Anjos MB Zuanon J (2009) Mechanisms of chromosomal evolution and its possible relation to natural history characteristics in *Ancistrus* catfishes (Siluriformes: Loricariidae). Journal of Fish Biology 75: 2209–2225. doi: 10.1111/j.1095-8649.2009.02450.x 20738683 10.1111/j.1095-8649.2009.02450.x

[B29] Pinkel D Straume T Gray JW (1986) Cytogenetic analysis using quantitative, high sensitivity, fluorescence hybridization. Proceedings of the National Academy of Sciences of the United States of America 83: 2934–2938. doi: 10.1073/pnas.83.9.2934 3458254 10.1073/pnas.83.9.2934 PMC323421

[B30] Reis DAR Brandão KO Toledo LFA Pazza R Kavalco KF (2012) Localização física dos genes ribossomais 5S e 18S em *Ancistrus* sp. (Loricariidae: Ancistrini) de Angra dos Reis/RJ, Bacia dos Rios Costeiros. Evolução e Conservação da Biodiversidade 3: 39–44. doi: 10.7902/3issecbvol1.2012n13

[B31] Schweizer D (1976) Reverse fluorescent chromosome banding with chromomycin and DAPI. Chromossoma 58: 307–324. doi: 10.1007/BF00292840 10.1007/BF00292840 137107

[B32] Sumner AT (1972) A simple technique for demonstrating centromeric heterochromation. Experimental Cell Research 75: 304–306. doi: 10.1016/0014-4827(72)90558-7 4117921 10.1016/0014-4827(72)90558-7

[B33] Traldi JB Vicari MR Blanco DR Martinez JF Artoni RF Moreira-Filho O (2012) First karyotype description of *Hypostomus iheringii* (Regan, 1908): a case of heterochromatic polymorphism. Comparative Cytogenetics 6: 115–125. doi: 10.3897/compcytogen.v6i2.2595 24260656 10.3897/CompCytogen.v6i2.2595 PMC3833790

